# Patient Outcomes and Rate of Intensive Care Unit Admissions Following Bariatric Surgery: A Retrospective Cohort Study of 775 Patients

**DOI:** 10.7759/cureus.49667

**Published:** 2023-11-29

**Authors:** Ahmed Alanzi, Faisal Alamannaei, Sara Abduljawad, Ameera Ghuloom, Fatema A Alahmed, Asem E Alzaidani, Mohamed F Almusaifer, Mohamed A Alanezi, Shahid Adeel

**Affiliations:** 1 Anesthesia and Critical Care, King Hamad University Hospital, Muharraq, BHR; 2 Anesthesia, King Hamad University Hospital, Muharraq, BHR; 3 General Practice, Arabian Gulf University, Manama, BHR; 4 General Practice, Ministry of Health - Bahrain, Manama, BHR; 5 Internal Medicine, King Hamad University Hospital, Muharraq, BHR; 6 General Practice, Ministry of Health Holdings, Manama, BHR; 7 General Practice, Ministry of Health, Manama, BHR

**Keywords:** sepsis, laparoscopic sleeve gastrectomy, patient outcomes, mortality, icu admissions, bariatric surgery

## Abstract

Background

The last two decades have seen a significant rise in obesity and its adverse consequences across the globe. Bariatric surgery has emerged as a widely employed therapeutic approach for weight reduction and alleviating the risk of obesity-related chronic diseases such as diabetes, cardiovascular diseases, and cancer. As bariatric procedures are gaining popularity, the complications associated with these procedures can not be ignored. This retrospective study aimed to investigate the incidence of intensive care unit (ICU) admissions following bariatric surgery and ICU-related mortality.

Methodology

This retrospective study conducted at King Hamad University Hospital, Bahrain evaluated the patient outcomes and the rate of ICU admissions following bariatric surgery between 2018 and 2022. Demographic data of the patients were extracted from electronic health records. The primary endpoint was ICU admission incidence and mortality, while secondary outcomes included risk factors, duration of ICU stay, and complications leading to ICU admission.

Results

Of the 775 patients included, 66.3% were female. The mean age of the patients was 35.92 ± 21.12 years. Over 91% of the patients had a body mass index above 30 kg/m^2^. The most common primary procedure was laparoscopic sleeve gastrectomy (75%), followed by gastric bypass (22.6%). In revision bariatric surgery, the majority (91.3%) had a conversion from sleeve gastrectomy to gastric bypass. Overall, 0.77% of patients were admitted to the ICU, with the majority being unplanned ICU admissions (0.52%). The average ICU stay was 21 days (1 to 54 days). The most common reason for ICU admission was sepsis, septic shock, and gastric leakage.

Conclusions

The results of this study show a relatively lower number of ICU admissions after bariatric surgery compared to previous studies.

## Introduction

Obesity has emerged as a global epidemic with far-reaching consequences for public health. Currently, the prevalence of obesity in the Middle East region is estimated at 21.17%, according to a systemic review of 101 studies with 698,905 participants published in 2022 [1]. In Bahrain, 39.5% of females and 28.4% of males are obese, which is much higher compared to the regional average of 10.3% for women and 7.5% for men [2]. As obesity is on the rise, an increasing volume of bariatric surgeries has also been reported in the Middle East region [3]. Bariatric surgery has emerged as an effective intervention to combat severe obesity and its associated health problems. It has also demonstrated efficacy in weight reduction and the incidence of other comorbidities such as diabetes, hypertension, and obstructive sleep apnea [4].

Based on the significant outcomes achieved with bariatric surgery, the number of people undergoing these procedures has increased tremendously. According to the International Federation for the Surgery of Obesity and Metabolic Disorders (IFSO) survey, almost 580,000 people undergo bariatric surgery annually in the world [5]. Although there are different types of bariatric procedures, there is no ideal procedure for each patient and all approaches are associated with some complications. According to the fourth report of the IFSO involving 394,431 surgical operations, 38.2% underwent Roux en Y gastric bypass, 46.0% sleeve gastrectomy, and 7.6% anastomosis gastric bypass bariatric surgery [6]. The gastric bypass with Roux-en-Y reconstruction has long been regarded as the preferred and highly effective bariatric method. However, vertical sleeve gastrectomy has also gained significant popularity in the last two decades owing to its efficacy and relative simplicity as a surgical approach. From 2003 to 2013, vertical sleeve gastrectomy increased from 0% to 37% [7]. Similarly, another study that analyzed bariatric surgeries from 1993 to 2016 reported that 60% of patients undergo vertical sleeve gastrectomy [8].

As the number of patients undergoing bariatric surgery is increasing, there has been an increase in revisional surgeries. Several reasons can prompt a revision of bariatric surgery, including weight gain, insufficient weight loss, or surgical complications. The incidence of revisional procedures after primary bariatric surgery is estimated at between 3% to 60% and is dependent on the primary bariatric procedure [9]. The incidence of revision procedures is 29%-39% in vertical banded gastroplasty [10], 10.5% and 60% for adjustable gastric banding [11], and 15% to 35% for gastric bypass [12]. Although bariatric procedures have demonstrated significant positive outcomes for obese patients, such surgical procedures are not without risks. Several studies have demonstrated postsurgical complications following bariatric surgery that require intensive care unit (ICU) admission [4,13]. The complications can be categorized into two groups, namely, early complications (<10 days) and late complications (>10 days). Early onset of complications leads to substantial negative health outcomes compared to delayed onset of complications. Among the early problems, the most significant ones encompass surgical wound infections, anastomosis dehiscence, fistulas, hemorrhage, venous thromboembolism, and incisional hernias [14]. The incidence of postoperative complications is rather low, with hemorrhage occurring in 0.5% of cases, thromboembolism in 0.8% of cases, and operational wound problems in 1.8% of cases [15,16]. The decision to admit patients to the ICU after bariatric surgery is controversial, with the majority of the decisions being made based on the surgeon’s experience. However, most researchers advocate the avoidance of ICU hospitalization to reduce costs and length of hospital stay [17].

Despite the significant amount of research across the globe, there is a paucity of data from the Middle East and North Africa region. Therefore, the present study was undertaken to assess the incidence of ICU admission following bariatric surgery at King Hamad University Hospital, Bahrain.

## Materials and methods

Study design and settings

The retrospective study was conducted at King Hamad University Hospital, Bahrain after obtaining approval from the institutional review board.

Study population

This study included all patients who underwent bariatric surgery between 2018 and 2020. The study also included patients who required ICU admission after the surgical procedure.

Data collection

The demographic data of the patients were retrieved from the electronic health records of the hospital. The demographic data included age, gender, weight, body mass index (BMI), surgical procedure, duration of the surgery, endotracheal tube size, ICU admission details, planned or unplanned ICU admission, and laboratory findings.

Study endpoints

The primary outcome of the study was to identify the incidence of ICU admission following bariatric surgery and ICU mortality in admitted patients. The secondary outcomes included risk factors for ICU admissions, the number of days of ICU admission, and medical complications that led to admission to the ICU.

Statistical analysis

Statistical analysis was conducted using SPSS version 20 (IBM Corp., Armonk, NY USA). To describe the demographic information of the participants, including age, gender, and occupation, descriptive statistics were utilized. Non-parametric data were analyzed using the Mann-Whitney U test, continuous data with the Student’s t-test, and categorical data with the Fisher’s exact test. Statistical significance was defined as a p-value less than 0.05. In accordance with the study questions and hypotheses, the results of the statistical analysis were presented in tables and graphs and analyzed.

## Results

A total of 775 patients were included in the analysis, with a majority of female patients (66.3%, n = 514). The BMI of patients was divided into various categories. The highest number of patients (26.8%, n = 208) had a BMI between 40 and 44.9 kg/m^2^, followed by ≥50 kg/m^2^ (21.5%, n = 167) and 45-49.9 kg/m^2^ (21.3%, n = 165). A total of 97.3% (n = 754) of patients had a BMI above 30 kg/m^2^. Regarding comorbidities, 50.58% (n = 392) of patients were suffering from hypertension and 26.45% (n = 205) from diabetes (Table 1).

**Table 1:** Demographic details of the patients. The data are presented as both N and %.

Variable		n	%
Gender			
	Female	514.00	66.30
	Male	261.00	33.70
BMI (kg/m^2^)			
	18.5–24.9	7.00	0.90
	25–29.9	14.00	1.80
	30–34.9	50.00	6.50
	35–39.9	164.00	21.20
	40–44.9	208.00	26.80
	45–49.9	165.00	21.30
	≥50	167.00	21.50
Comorbidities			
	Obstructive sleep apnea	117	15.09
	Ischemic heart disease	197	25.41
	Hypertension	392	50.58
	Diabetes mellitus	205	26.45

The mean age of the patients was 35.92 ± 21.12 years. The mean height and weight of the patients were 165.45 ± 9.94 cm and 122.57 ± 28.1 lb, respectively (Table 2).

**Table 2:** Demographic details of the patients’ age, height, and weight. Demographic variables are presented as mean ± SD.

Variable	Mean ± SD
Age (years)	35.92 ± 21.12
Height (cm)	165.45 ± 9.94
Weight (lbs)	122.57 ± 28.1

**Figure 1:**
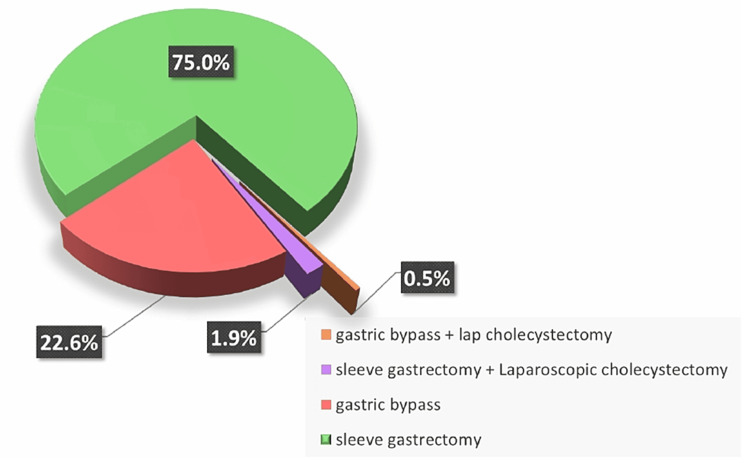
Types and percentages of primary procedures in patients. The data are presented as %.

The majority of the participants underwent laparoscopic sleeve gastrectomy (75%, n = 564), followed by gastric bypass surgery (22.6%, n = 170), sleeve gastrectomy and laparoscopic cholecystectomy (1.9%, n = 14), and gastric bypass plus laparoscopic cholecystectomy (0.5%, n = 4) (Figure 1).

**Figure 2:**
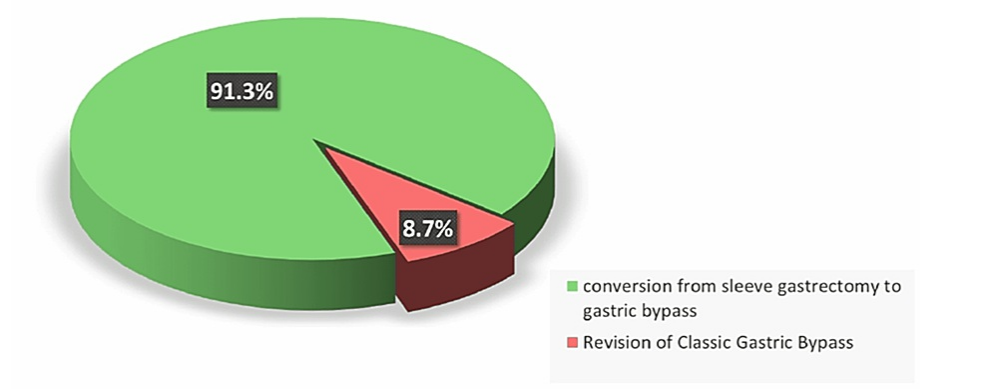
Types of revision procedures and percentages performed on patients. The data are presented as %.

Overall, 91.3% (n = 21) of patients underwent conversion from sleeve gastrectomy to gastric bypass, whereas 8.7% (n = 2) of patients underwent a revision of classic gastric bypass (Figure 2).

Table 3 describes the type and duration of the procedure. Overall, 752 (97%) procedures were primary bariatric surgery and 23 (3%) were revisional bariatric surgery. The majority of the procedures were conducted in 2018, with primary (99.7%, n = 299) and revision (0.3%, n = 1). In 2019, all procedures were primary whereas in 2020, 91.1% (n = 224) of bariatric procedures were primary.

**Table 3:** Types and durations of the procedures. The frequency of types of procedures is presented as N and %. The duration of procedures is presented as mean ± SD. Mean in minutes.

Year	Type of procedure	Name of procedure	n	%	Duration of procedure (mean ± SD)
2018	Primary n (%) = 299 (99.7%)	Sleeve gastrectomy	268.00	89.63	79.55 ± 17.8
		Sleeve gastrectomy + Laparoscopic cholecystectomy	4.00	1.34	75 ± 17.32
		gastric bypass	27.00	9.03	90 ± 26.31
	Revision n (%) = 1 (0.3%)	Conversion from sleeve gastrectomy to gastric bypass	1.00	100.0	120.00
2019	Primary n (%) = 229 (100%)	Sleeve gastrectomy	184.00	80.35	50.52 ± 16.06
		Sleeve gastrectomy + Laparoscopic cholecystectomy	2.00	0.87	60 ± 0
		Gastric bypass	43.00	18.78	64.88 ± 33.34
	Revision n (%) = 0 (0.0%)		0.00	0.00	
2020	Primary n (%) = 224 (91.1%)	Sleeve gastrectomy	112.00	50.00	63.13 ± 11.45
		Sleeve gastrectomy + Laparoscopic cholecystectomy	8.00	3.57	78.75 ± 22.32
		Gastric bypass	100.00	44.64	71.2 ± 19.84
		Gastric bypass + lap cholecystectomy	4.00	1.79	67.5 ± 15
	Revision n (%) = 22 (8.9%)	Conversion from sleeve gastrectomy to gastric bypass	20.00	90.91	70.5 ± 20.12
		Revision of classic gastric bypass	2.00	9.09	75 ± 21.21

In 2018, only one (0.13%) patient was admitted to the ICU after the surgery. In 2020, five (0.64%) patients had ICU admission after bariatric surgery. ICU-related mortality occurred in two (0.26%) patients (Table 4).

**Table 4:** Intensive care unit (ICU) admissions following bariatric surgery. The data are presented as n and %.

	ICU planned		ICU unplanned		ICU total		Mortality	
Year	n	%	n	%	n	%	n	%
2018	0.00	0.00	1.00	0.13	1.00	0.13	0.00	0.00
2019	0.00	0.00	0.00	0.00	0.00	0.00	0.00	0.00
2020	2.00	0.26	3.00	0.39	5.00	0.65	2.00	0.26
total	2.00	0.26	4.00	0.52	6.00	0.77	2.00	0.26

Table 5 describes the details of each patient who was admitted to the ICU after bariatric surgery. Three patients underwent primary bariatric surgery, whereas three patients underwent revision surgery. The number of ICU days ranged from 1 to 54 days. The most common reason for admission was sepsis and septic shock (Table 5).

**Table 5:** Individual details of patients who underwent intensive care unit (ICU) admission after bariatric surgery. OSA: obstructive sleep apnea; ARDS: acute respiratory distress syndrome; CPAP: continuous positive airway pressure; LFTs: liver function tests; POD: postoperative day; BMI: body mass index

Age	Gender	BMI (kg/m^2^)	Name of procedure	Primary or *revision	Duration of operation (minutes)	Planned/Unplanned	How many days in the ICU?	Reason of admission
52	F	38.97	Sleeve gastrectomy	Revision	120	Unplanned (POD 7)	54	Septic shock, ARDS
44	M	55	Single anastomosis gastric bypass	Primary	90	Planned	1	OSA, to continue on CPAP
38	M	70	Sleeve gastrectomy	Primary	60	Unplanned (POD 8)	30	Septic shock, mesenteric ischemia, hypernatremia, elevated LFTs, pancreatitis
35	F	36.6	Conversion of sleeve gastrectomy to mini gastric bypass	Revision	90	Unplanned (POD 7)	10	Sepsis, gastric leakage
53	F	74	Gastric bypass	Primary	90	Planned	25	Sepsis, gastric leakage
30	M	65	Gastric bypass	Primary	60	Unplanned (POD 9)	5	Upper gastrointestinal bleeding

## Discussion

This retrospective study showed that only a small number of patients (0.77%) required ICU admission following bariatric surgery. These are significant findings as previous studies have reported much higher ICU support after undergoing bariatric surgery [18,19]. A study by Helling et al. reported an ICU admission rate of 24% in 250 patients who underwent either vertical banded gastroplasty or Roux-en-Y gastric bypass surgery [19]. Furthermore, they also revealed that male gender, BMI over 60 kg/m^2^, and age above 50 years were associated with ICU admission [19]. Similarly, Morgan et al. in their study of 12,062 bariatric surgeries, with a follow-up of at least 12 months and up to 3.4 years, reported that 590 (4.9%) required admission to the ICU after surgery. Furthermore, out of 4.9% of ICU admissions, 30.9% were unplanned referrals [13]. In the present study, the majority (66.7%) of the ICU admissions were unplanned. Morgan et al.also reported that the major reasons for ICU admission were associated with surgical procedures (89.0%), anesthesia (8.8%), and medical complications (2.2%) [13].

Similarly, Froylich et al. conducted a retrospective analysis of all ICU admissions in patients who had undergone bariatric surgery between 2006 and 2013 [20]. The study revealed that 124 out of 4,398 (2.8%) patients required ICU admission following the surgery. Respiratory failure was the most frequent cause of ICU admission, affecting 35 (28.2%) patients. The average duration of ICU stay was 6.0 ± 9.6 days. Mortality was observed in five (4.0%) patients [20]. Some studies have shown much higher rates of ICU admissions after bariatric surgery. A study by Penna et al.reported that out of 828 patients, 38.5% were admitted to the ICU after surgery [18]. In the present study, sepsis and septic shock were the most common reasons for ICU admission. The most common reason for sepsis is anastomotic leak which leaks into the abdominal cavity, resulting in peritonitis and sepsis. Following bariatric surgery, the development of sepsis has been attributed to an increased risk of mortality [21]. Kelles et al. reported that 42.7% of deaths after bariatric surgery were due to sepsis [22]. Similarly, Kermarrec et al. reported that sepsis patients with preoperative weight (BMI >50 kg/m^2^) and those who underwent multiple reoperations had a worse prognosis in the ICU [23].

Another common concern in obese patients after bariatric surgery is respiratory complications. In the present study, only one out of six ICU-admitted patients developed respiratory complications. Adverse respiratory events are approximately reported in 2% of the patients after bariatric surgery [24]. As the number of ICU admissions was very small in this study, our findings do not clearly show the various factors that lead to ICU admissions as reported by previous studies. Regarding gender distribution, our study had a higher percentage of female patients (66.30%) compared to males (33.70%). This is consistent with the existing literature, which often reports a higher prevalence of obesity and, consequently, a greater demand for bariatric surgery among females. The Nationwide Inpatient Sample database was reviewed for obese patients undergoing bariatric surgery between 2002 and 2011. During the 10-year period, 810,999 patients underwent bariatric surgery; 19.3% were male and 80.7% were female [25].

Laparoscopic sleeve gastrectomy was the most common primary procedure (75%), followed by gastric bypass surgery (22.6%). This distribution is consistent with current clinical practice, where laparoscopic sleeve gastrectomy and gastric bypass are widely accepted as effective bariatric procedures [26,27]. Conversion from sleeve gastrectomy to gastric bypass was the most common revision procedure (91.3%). Gastric bypass is considered the procedure of choice for revision bariatric surgery due to its effectiveness in weight reduction and safety [28]. ICU-related mortality was observed in two out of six patients, representing a mortality rate of 0.33%. Mortality following bariatric surgery is a critical concern and is often attributed to various factors, including the complexity of the surgical procedure, the overall health of the patient, and the presence of comorbid conditions. Bariatric surgery-related mortality is usually reported below 1%, with most studies reporting 0.3% [15,18].

The present study has some limitations that should be acknowledged before interpreting the findings. As the study focused on a particular healthcare facility, this may limit the generalizability of our findings to broader populations or settings. The lower percentage of ICU admissions following bariatric surgery is an important finding that should be validated with prospective studies with larger sample sizes. Additionally, long-term follow-up of patients is essential to assess the sustainability of weight loss and the impact on comorbidities beyond the immediate postoperative period.

## Conclusions

The present study reported a much lower rate of ICU admissions after bariatric surgery compared to previous studies. Apart from this, most of our findings were consistent with published literature. These findings highlight the need for comprehensive preoperative evaluations and individualized treatment plans to optimize the safety and success of bariatric surgery.
